# Evidence for sexual conflict over major histocompatibility complex diversity in a wild songbird

**DOI:** 10.1098/rspb.2018.0841

**Published:** 2018-08-01

**Authors:** Jacob Roved, Bengt Hansson, Maja Tarka, Dennis Hasselquist, Helena Westerdahl

**Affiliations:** Department of Biology, Molecular Ecology and Evolution Lab, Lund University, Ecology Building, 223 62 Lund, Sweden

**Keywords:** major histocompatibility complex, great reed warbler, cost of immune responses, sexually antagonistic selection, immunopathology, MHC diversity

## Abstract

Sex differences in parasite load and immune responses are found across a wide range of animals, with females generally having lower parasite loads and stronger immune responses than males. Intrigued by these general patterns, we investigated if there was any sign of sex-specific selection on an essential component of adaptive immunity that is known to affect fitness, the major histocompatibility complex class I (MHC-I) genes, in a 20-year study of great reed warblers. Our analyses on fitness related to MHC-I diversity showed a highly significant interaction between MHC-I diversity and sex, where males with higher, and females with lower, MHC-I diversity were more successful in recruiting offspring. Importantly, mean MHC-I diversity did not differ between males and females, and consequently neither sex reached its MHC-I fitness optimum. Thus, there is an unresolved genetic sexual conflict over MHC-I diversity in great reed warblers. Selection from pathogens is known to maintain MHC diversity, but previous theory ignores that the immune environments are considerably different in males and females. Our results suggest that sexually antagonistic selection is an important, previously neglected, force in the evolution of vertebrate adaptive immunity, and have implications for evolutionary understanding of costs of immune responses and autoimmune diseases.

## 1. Introduction

Females and males differ in the strength and nature of immune responses and these differences are well established across a wide range of animals [1–3]. Males are parasitized more often than females [4,5] and this has been attributed to sex differences in both immune system capabilities to fight off pathogens and in parasite exposure [1,3,5]. Females often have stronger immune responses than males [2,3,6,7]; however, stronger immune responses are not always beneficial in terms of physiological condition and Darwinian fitness (hereafter called fitness) [8–10]. Sex differences in adaptive immune responses and their evolutionary consequences have mostly been studied in mammals, but also in birds [2,11]. The general finding among mammals is that females tend to be more susceptible to immunopathology (i.e. immune response-induced damage to own tissue, such as autoimmunity), compared to males [3,7]. Moreover, in both humans and mice, susceptibility to autoimmune disease is found to be associated with genetic components of adaptive immunity, in particular gene variants (alleles) at the major histocompatibility complex (MHC) [7,8,12,13].

MHC molecules are central in the adaptive immune response of vertebrates, and they are encoded by a set of genes that are highly polymorphic [14]. The exceptionally high polymorphism of MHC genes (i.e. number of different alleles) is considered to be maintained by selection from pathogens [14–16]. The MHC has two main classes involved in presentation of peptide antigens, MHC class I and II, and both classes contain duplicated genes [14]. The number of MHC gene copies differs vastly between species and may also (to a lesser degree) vary between individuals within a species. High MHC diversity has been hypothesized to be beneficial either because carrying many different MHC alleles allows the immune system to recognize a broader range of pathogens [17–19], or because having a high MHC diversity increases the chance of carrying specific advantageous alleles that maximize disease resistance [20,21]. However, too high MHC diversity has been hypothesized to be disadvantageous, either due to increased risk of immunopathology or because it diminishes the antigen-recognition repertoire of T-cells (due to stronger negative selection of T-cells in the thymus) [14,22]. Thus, theory and empirical data predict a trade-off and hence optimal levels of MHC diversity [22–24].

Recently, it was hypothesized that sex differences in the strength of immune responses may result in different optimal levels of MHC diversity in males and females, and thus create a basis for sexually antagonistic selection [6]. Sex differences in immune responses have previously been overlooked when studying natural selection in ecology and evolution. The hypothesis of sexually antagonistic selection on immunity genes offers a promising new research avenue to be investigated across a wide range of species. According to sexual conflict theory, a genetic (intra-locus) sexual conflict arises when males and females have different fitness optima for a trait, and at the same time share the genetic background for that trait [25–27]. MHC genes are duplicated, tightly linked and located on autosomes, and previous studies have shown that variation in MHC diversity can affect adaptive immune responses [28–30]. Thus, the basic conditions for a genetic sexual conflict are at hand: (a) MHC is linked to fitness and may be subject to sexually antagonistic selection and (b) males and females share the genetic architecture of the MHC [31–33].

The concept of genetic sexual conflict has been widely studied [34], also in wild populations [35–38], but previous studies have largely focused on phenotypic traits (in empirical studies, often sexually dimorphic traits) or on genome-wide fitness effects (where the involved traits and genes are not known) [26,27]. Our study presents a novel approach in that we investigate sexual conflict on a single multigene immune locus (i.e. a genomic region containing several tightly linked duplicated MHC gene copies (J Roved, M Strandh 2018, unpublished data; [39])) that is known to have a strong impact on fitness.

We investigate sex differences in the effects of MHC diversity on key components of fitness using samples and detailed data from a 20-year study of a local population of a songbird, the great reed warbler *Acrocephalus arundinaceus*, in Sweden [37,40,41]. Long-term studies of songbirds offer excellent opportunities to investigate the effects of MHC diversity in the wild [42], because songbirds have large numbers of MHC gene copies [19,43], are subject to natural selection from pathogens [39,43], and fitness components can be estimated at the individual level over the entire lives of the birds [17,44]. Previous studies of our great reed warbler study population revealed associations between MHC class I (MHC-I) alleles and avian malaria [21,45], and signs of balancing selection on MHC-I [39,46]. Here, we analyse the effects of MHC-I diversity (i.e. the number of different MHC-I alleles per individual) and sex on three independent components of fitness in male and female great reed warblers: *life span*, *offspring fledging success* (i.e. an individual's lifetime number of fledglings accounting for its life span in the model), and *offspring recruitment success* (i.e. an individual's lifetime number of offspring that returned to breed in the study area accounting for its lifetime number of fledglings in the model).

## 2. Results

MHC-I diversity (i.e. the number of different MHC-I alleles per individual) did not differ between the sexes in our dataset, neither among adult nor nestling great reed warblers (mean number of different MHC-I alleles in adults; males 13.6 (s.d. = 3.1) and females 13.5 (s.d. = 3.1), *t*-test: *t* = 0.32, d.f. = 182.12, *p* = 0.75, *N* = 88 males and 100 females; electronic supplementary material, figure S1*a*; mean number of different MHC-I alleles in nestlings; males 13.3 (s.d. = 3.2) and females 13.5 (s.d. = 3.0), *t*-test: *t* = −0.54, d.f. = 133.54, *p* = 0.59, *N* = 84 males and 61 females; electronic supplementary material, figure S1*b*).

When we modelled the effects of MHC-I diversity on *life span* and on *offspring fledging success*, we found no significant effects of MHC-I diversity, nor any significant interactions between MHC-I diversity and sex (electronic supplementary material, Results, table S1d, fig. S2, table S2c, fig. S3).

**Figure 1.**
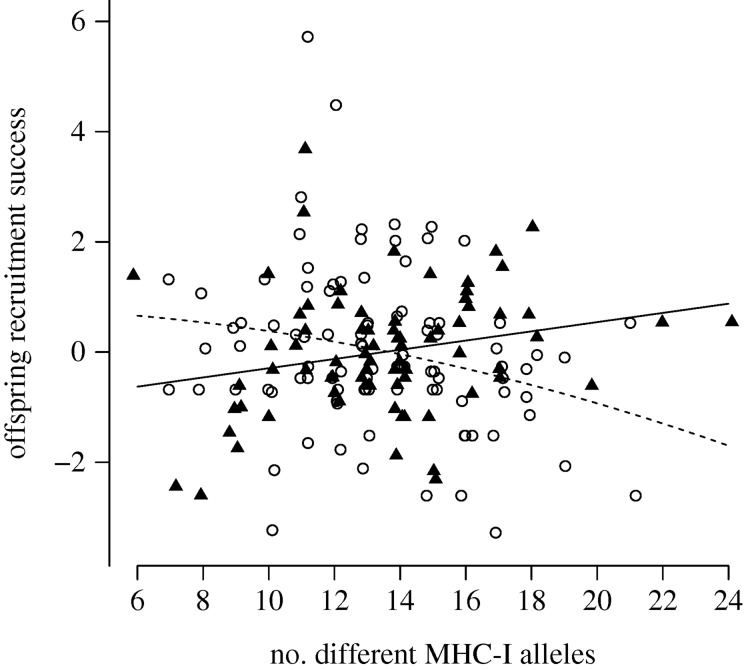
The relationship between MHC-I diversity (i.e. the number of different MHC-I alleles per individual) and *offspring recruitment success*. Females are shown with open circles and males are shown with black triangles. The lines show the predictions from regression models of *offspring recruitment success* on MHC-I diversity in females (broken line) and males (solid line) (see the text for model details). *Offspring recruitment success* is illustrated using the residual lifetime number of recruiting offspring from a regression with lifetime number of fledglings. Removing the two outliers with high *offspring recruitment success* in the females did not affect the model predictions. Note: jitter was added to the number of different MHC-I alleles to distinguish individual data points.

When testing the effect of MHC-I diversity on *offspring recruitment success*, we found a significant interaction between MHC-I diversity squared and sex (quadratic regression, *b* = 0.0071, *p* = 0.0028; figure 1; table 1). Owing to the significant interaction between sex and MHC-I diversity on *offspring recruitment success*, we repeated this analysis for each sex separately. In males, the relationship between MHC-I diversity and *offspring recruitment success* was significantly positive (linear regression, *b* = 0.084, *p* = 0.045; figure 1; table 1). By contrast, in females this relationship was significantly negative (quadratic regression, *b* = −0.0044, *p* = 0.018; figure 1; table 1). These results suggest that sexually antagonistic selection on MHC-I diversity partly explains *offspring recruitment success*. Within each sex, the average effect of having one more MHC-I allele was a gain of 0.084 offspring recruits in males, while it was a loss of 0.111 offspring recruits in females (estimated by linear approximation). The standard deviation (s.d.) of MHC-I diversity was 3.0 alleles in females and 3.2 in males, and a change of 1 s.d. thus corresponds to a difference of approximately 0.6 lifetime offspring recruits between an average male and an average female. In comparison, the mean lifetime number of offspring recruits in our great reed warbler study population was 1.90 (s.d. = 2.10, *N* = 77 adult males and 96 adult females). Estimates of selection gradients for the effects of MHC-I diversity on *offspring recruitment success* are given in electronic supplementary material, table S4.

**Table 1.** Results from regression models of the effects of MHC-I diversity (i.e. the number of different MHC-I alleles per individual) and sex on *offspring recruitment success* for both sexes, as well as males and females separately.

	estimate	s.d.	*t*-value	*p*-value
*both sexes*				
(intercept)	0.42	0.38	1.13	0.26
fledglings	0.21	0.019	11.37	<0.0001
(MHC-I diversity)^2^	−0.0044	0.0017	−2.65	0.0088
sex (male)	−0.93	0.55	−1.67	0.10
fledglings × sex (male)	−0.069	0.022	−3.08	0.0025
(MHC-I diversity)^2^ × sex (male)	0.0071	0.0023	3.04	0.0028
*females*				
(intercept)	0.42	0.41	1.03	0.31
fledglings	0.21	0.021	10.34	<0.0001
(MHC-I diversity)^2^	−0.0044	0.0018	−2.41	0.018
*males*				
(intercept)	−1.12	0.60	−1.85	0.068
fledglings	0.14	0.011	13.39	<0.0001
MHC-I diversity	0.084	0.041	2.04	0.045

**Figure 2.**
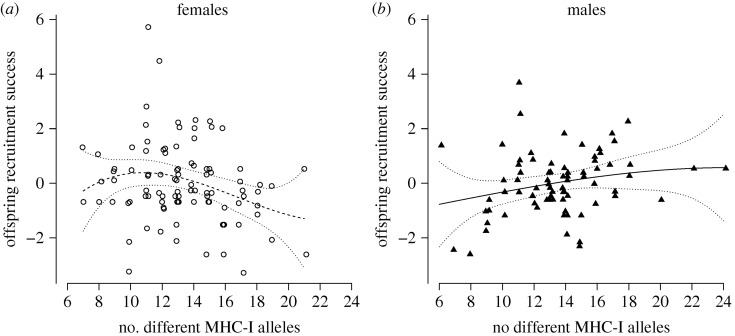
The relationship between the MHC-I diversity (i.e. the number of different MHC-I alleles per individual) and *offspring recruitment success* in females (*a*) and males (*b*) showing predictions (±2 s.e.) from cubic spline models (see the text for model details). *Offspring recruitment success* is here illustrated using the residual lifetime number of recruiting offspring from a regression with lifetime number of fledglings. Removing the two outliers with high *offspring recruitment success* in the females did not affect the predictions of the cubic spline model. Note: jitter was added to the number of different MHC-I alleles to distinguish individual data points.

Our models did not show evidence for optimal MHC-I diversity on any of the fitness components (*life span*, *offspring fledging success*, and *offspring recruitment success*) within the observed range of MHC-I diversity. However, sex-specific optima towards the edges of the observed range may be difficult to detect due to the low numbers of individuals that have very low or very high MHC-I diversity. We, therefore, visualized the association between *offspring recruitment success* and MHC-I diversity using cubic spline models (note that these analyses were based on the residual lifetime number of recruiting offspring from a regression with lifetime number of fledglings, see Methods). The results from these models indicate (but note, no significant support) that females have an optimum around 10 different MHC-I alleles, while the optimum for males appears to be much higher (possibly >20 alleles; figure 2). These tentative female and male optima fall on either side of the population mean of 13.5 different MHC-I alleles per individual.

Disassortative mating, in the sense that females with few MHC alleles prefer to mate with males with many MHC alleles and vice versa, could have caused a spurious negative correlation between MHC-I diversity in males and females, and thus led to a false conclusion about sexually antagonistic selection working through *offspring recruitment success* [47]. However, we found no correlation between MHC-I diversity of social pair mates (Pearson's correlation, *r* = 0.043, *N* = 111 mated pairs, *t* = 0.45, d.f. = 109, *p* = 0.65). Another factor that could have confounded our results of sexually antagonistic selection on parental MHC-I diversity is the effect of the offspring's own MHC-I diversity on their recruitment success. The MHC-I diversity was significantly correlated between offspring and their parents (generalized linear mixed model, *b* = 0.393, s.e. = 0.042, *t* = 9.47, *p* < 0.0001; electronic supplementary material, table S5). Note that the *p*-value for this model was approximated (see electronic supplementary material, Methods for details). However, when testing the effect of MHC-I diversity on recruitment status in the offspring from the 1998 cohort, we found no significant effect (GLMM, *b* = −0.087, *p* = 0.19; electronic supplementary material, table S6*a*), and there was no interaction between MHC-I diversity and sex of the offspring that influenced whether they became recruits or not (GLMM, *b* = 0.091, *p* = 0.52; electronic supplementary material, table S6*b*). These results suggest that the effect of the parents' MHC-I diversity on *offspring recruitment success* is not a consequence of the MHC-I diversity in the offspring *per se*.

**Figure 3.**
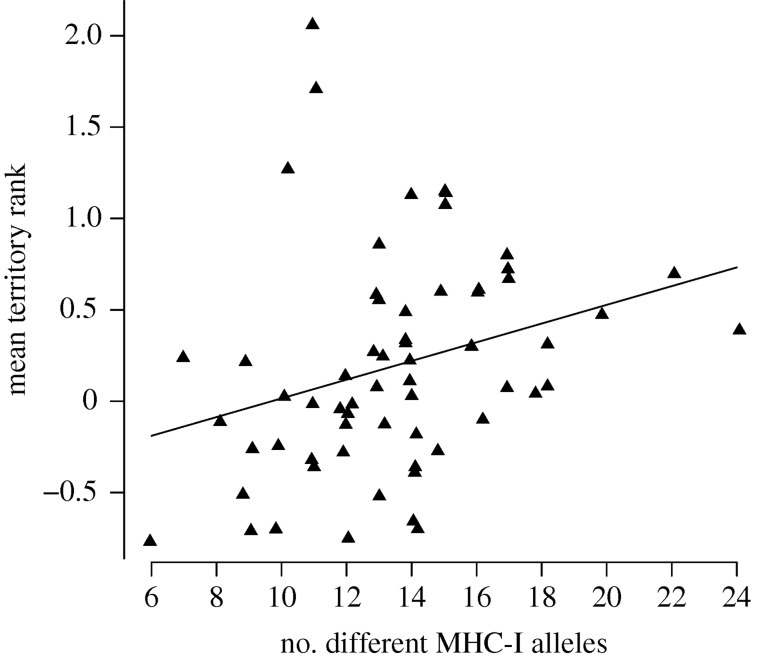
The relationship between the mean territory rank and MHC-I diversity (i.e. the number of different MHC-I alleles per individual) in males with predicted values from a linear regression model (see the text for model details). The relationship is illustrated using the lifetime mean of age-standardized territory attractiveness rank values (see electronic supplementary information). Note: jitter was added to the number of different MHC-I alleles to distinguish individual data points.

Non-genetic parental factors could also influence our results on sexually antagonistic selection on MHC-I diversity in males and females. In previous studies of our great reed warbler study population, the quality of the parents' territory has been shown to have a positive effect on the lifetime number of fledglings as well as on *offspring fledging success* [41,48,49]. We, therefore, investigated if territory attractiveness was related to MHC-I diversity in parents. In males, there was a significant positive relationship between territory attractiveness rank and MHC-I diversity (LMM, *b* = 0.061, s.e. = 0.019, *t* = 3.30, *p* < 0.0016; figure 3; table 2), while no such relationship was found in females (LMM, *b* = 0.002, s.e. = 0.022, *t* = 0.10, *p* = 0.92; electronic supplementary material, fig. S4; table 2). Note that the *p*-values for these models were approximated (see electronic supplementary material, Methods for details).

**Table 2.** Results from linear mixed effects models of the effects of MHC-I diversity (i.e. the number of different MHC-I alleles per individual) on the territory attractiveness rank in males (*N* = 63) and females (*N* = 81), respectively.

	estimate	s.e.	*t*-value	*p*-value
*males*				
(intercept)	−0.548	0.261	−2.10	0.040
MHC-I diversity	0.061	0.019	3.30	0.0016
*females*				
(intercept)	0.335	0.303	1.11	0.27
MHC-I diversity	0.0021	0.022	0.10	0.92

## 3. Discussion

In this study, we found signatures of sexually antagonistic selection acting on MHC-I diversity (i.e. the number of different MHC-I alleles per individual) in a wild population of great reed warblers, although the MHC-I diversity in males and females did not differ. Sexually antagonistic selection acting on MHC-I diversity is a novel finding in the context of sexual conflict, as it presents the first-ever empirical evidence for an unresolved genetic sexual conflict on an immune gene locus (here, the compact and tightly linked MHC-I multigene locus) [26,27]. In terms of *offspring recruitment success*, we found that selection on MHC-I diversity acted in opposite directions in adult males and females (figure 1). Females would have gained from having a lower MHC-I diversity, while males would have gained from having a higher MHC-I diversity. This sexual conflict over MHC-I diversity cannot be solved on the genomic level, because the MHC genes are autosomal and thus shared between males and females [31–33]. Theoretical and empirical work states that pathogen diversity shapes and maintains MHC diversity [15,16,50]; however, previous theory does not consider the fact that the immune environments are considerably different in males and females (e.g. due to effects of sex hormones on immune responsiveness). Our results are in accordance with the ideas that sexually antagonistic selection is an important force generating and maintaining genetic variation in populations in general [35,51], and even in mediating selection for genetic variation in disease resistance [52]. Our results also support the recent hypothesis that sex differences can be a key component to better understand the selective forces that maintain and constrain MHC diversity [6].

We did not observe any effect of the parents' MHC-I diversity on *offspring fledging success*, thus MHC-I diversity does not seem to have a strong effect on offspring quality at the nestling stage. Instead, our data suggest that the MHC-I diversity of the parents acts through delayed effects that influence post-fledging survival of the offspring until time of recruitment (≥1 year of age), i.e. *offspring recruitment success*. This effect on *offspring recruitment success* was not explained by the MHC-I diversity of the offspring themselves, but solely by the MHC-I diversity of the parents. We, therefore, conclude that the effect of the parents' MHC-I diversity on *offspring recruitment success* is a phenotypic effect of the parents rather than a heritable effect acting through the offspring genotype.

The mechanisms that mediate the effect of the parents' MHC-I diversity on *offspring recruitment success* need to be investigated further. However, a possible explanation is that male parents with high and female parents with low MHC-I diversity are in better physiological condition, allowing them to provide better parental care which results in offspring of higher quality, i.e. offspring that are more likely to become recruits. The positive correlation between MHC-I diversity and territory attractiveness rank in males (figure 3), further explains the link between *offspring recruitment success* and MHC-I diversity in males. If males with high MHC-I diversity on average occupy better territories, they are able to provide the fledglings with more (or better) resources [49], and thereby increase *offspring recruitment success*. The fact that we observe an effect on offspring recruitment success but not on offspring fledging success may be caused by differences in selection during the nestling and juvenile stages. Whether a nestling fledge or not may be mainly affected by factors such as food provision and nest predation [41,48,49], while offspring quality may not be truly advantageous until the offspring meet the stresses of life outside the nest, e.g. independent foraging, predator avoidance, migration, and notably pathogen exposure in tropical winter quarters. We found no correlation between MHC-I diversity and territory attractiveness rank in females. One factor that could explain the lack of such a correlation is that territory quality is less likely to be associated with physical condition in females compared to males. Females arrive later than males to the breeding grounds and evaluate the quality of the territory of potential mates, but also take into account the rank that she will get in the harem of the socially polygynous males, as well as the quality of their song [41,48,53]. For example, if a female settles with a male in a high-ranked territory, she is likely to obtain less help with parental care because such a male will have a larger harem [48,54]. Thus, the breeding site/mate choice decision process in the females is more complex than the straightforward competition for territories among males, and this is likely to weaken the association between physical condition and territory quality [41].

What property of the MHC-I genes could potentially cause the negative effects of high MHC-I diversity on *offspring recruitment success* in females? Individuals with high MHC diversity are supposedly at an advantage because they are able to detect more pathogens [17,18], but they also have a higher risk of carrying MHC alleles that may induce immunopathology, including autoimmunity [7,12,55,56]. Moreover, a recent study in humans showed that the usage of T-cells differs between men and women [57]. In humans, women are at a higher risk than men of contracting autoimmune diseases, and immune-regulating effects of sex hormones have been thought to be one factor that mediates these sex differences [58–60]. The similarity of avian and mammalian immune systems makes it likely that the risk of autoimmune diseases may be skewed in a similar way in birds [61]. Testosterone generally suppresses (directly or indirectly) the responsiveness of the vertebrate immune system [2,62], while female sex hormones (oestrogen, progesterone) generally enhance immune responsiveness [2,6,63]. Each MHC allele encodes an MHC molecule with certain antigen-binding properties and a predisposition for immunopathology [58,64], and due to sex hormone-induced differences in immune responsiveness, specific MHC molecules could be more likely to trigger immunopathological reactions in females than in males [6]. The overall weaker immune responses of males make immunopathological reactions less likely, and thus males may benefit from the advantage of increased MHC diversity (i.e. by being able to recognize more pathogens) without paying the costs of immunopathology.

We find it unlikely that great reed warbler females should benefit from having as few MHC-I alleles as possible, as suggested by the correlation with *offspring recruitment success* in our model (figure 1). Instead, we envision that there is an optimal level of MHC-I diversity and that this optimal level differs between the sexes, as suggested by the cubic spline visualization (figure 2). Although our statistical models show no evidence for optimal MHC-I diversity within its observed range, the cubic spline visualization indicates a tendency that the diversity optima for females and males are disparate, as expected for an unresolved sexual conflict. In relation to the population average in MHC-I diversity, males would benefit from having a higher number of different MHC-I alleles and females from having a lower number of different MHC-I alleles. In theory, traits under sexually antagonistic selection will ultimately evolve sexual dimorphism that will resolve the genetic sexual conflict; however, we find no sexual dimorphism in the number of different MHC-I alleles in great read warblers. This is expected as the MHC-I genes are autosomal [31–33], and traits with a shared genetic architecture between the sexes will be limited in their ability to evolve sexual dimorphism at the genomic level [25,65–67]. A sexual conflict may also be solved by alterations in gene expression, however our results indicate that such compensatory mechanisms have not evolved to an extent that resolves the sexually antagonistic selection on MHC-I diversity in the great reed warblers.

The divergent effects of male and female sex hormones on immune responses seem to be conserved across taxa [2,3,59], implying that sex differences in immune responsiveness are common among vertebrates [6]. In birds, sex hormone-induced differences in immune responses between males and females may be particularly pronounced during the breeding season [11], when the levels of circulating sex hormones are at their highest [62]. The fact that our study did not find any effect of the MHC-I diversity of the offspring on their recruitment success goes in line with this reasoning, and an explanation for this may be that hormone-mediated sexually antagonistic effects should only occur after the sex hormone levels increase at the onset of sexual maturity. In females, the physical effort of breeding in combination with high levels of female sex hormones that increase the immune responsiveness may increase the risk of immunopathological reactions, and particularly so in females with high MHC-I diversity [9]. Such immunopathological reactions may reduce the physiological condition and thus affect the ability of a female to provide resources to her offspring. Female songbirds make the most important contributions to offspring phenotype during the embryo [68,69] and the nestling stages [54,70]. Physiological factors that directly affect female condition during this time (such as costs and benefits of immune responses), may have a strong impact on offspring quality and the probability of post-fledging survival (and thus *offspring recruitment success*). Phenotypic costs caused by vaccination-induced immune system activation have previously been shown to decrease the feeding rate of female blue tits (*Cyanistes caeruleus*) and pied flycatchers (*Ficedula hypoleuca*) [71,72]. In female pied flycatchers, reduced feeding rates resulted in reduced offspring body mass and reduced fledging success (although the latter effect was absent in birds breeding under environmental stress conditions) [71]. Great reed warbler males, contradictory to females, gain *offspring recruitment success* from higher MHC-I diversity, and this may be a result of better protection against pathogens, allowing these males to arrive early and obtain attractive, high quality territories [41], thus providing resources that improve offspring development, physiological condition and survival to breeding age.

## 4. Conclusion

In the present study, we find support for sexually antagonistic selection acting on MHC-I diversity in great reed warblers. It is a remarkable result in the sense that certain levels of antigen-recognition ability (i.e. MHC-I diversity) are shown to be beneficial to one sex but disadvantageous to the other, and we propose how this sexually antagonistic selection is likely to be driven by a combination of differences in physiology and life histories between males and females. This empirical result supports recent hypotheses regarding the effects of sex and sex hormones on immune responsiveness and disease genetics [6,52].

Evidence of genetic sexual conflicts in wild populations is rare. Sexual conflict studies have traditionally focused on either specific phenotypic traits or on genome-wide fitness effects, where, it is unknown which specific genes that mediate the effects. In the present study, we examined fitness effects of genetic diversity in a specific immune gene complex (MHC-I) that is central in adaptive immunity and shared between the sexes. Our results show that the diversity in these genes is subject to sexually antagonistic selection, while we observe no difference in mean MHC-I diversity between the sexes, strongly suggesting an unresolved sexual conflict. We anticipate that sexual conflicts related to MHC genes, such as the one we have detected in the present study, may be common among vertebrates, because both the overall architecture of the immune system and the sex hormone effects on immune responsiveness are phylogenetically conserved.

We propose that the general topic of sexual conflict over immune gene diversity should be investigated more widely across vertebrates to gain insights into which properties of the immune genes are key to understand costs and benefits of immune responses and to elucidate why non-adaptive immunopathological reactions occur. Moreover, such research may unravel specific trade-offs related to MHC diversity, i.e. which factors select for high MHC diversity on the one side, and which factors counteract high MHC diversity on the other. Sexual conflicts over immunity may play a more important role in the evolution of the vertebrate immune system than previously assumed, and could even potentially help explain the over-representation of immunopathology in females versus males, e.g. in autoimmune diseases in humans [60].

## 5. Methods

The great reed warbler is a long-distance migratory songbird that breeds in Europe and western Asia between April and July and winters in sub-Saharan Africa [73]. It has a facultative polygynous social mating system with up to 4–5 females in a male's harem ([48] and unpublished data). In this study, we used data on 88 adult males and 100 adult females from a long-term study of individually colour-ringed great reed warblers at Lake Kvismaren (59°10′ N, 15°25′ E) in southern central Sweden 1984–2004 [37,40,41]. In this population, we have followed the reproductive success of each individual every year, which provides detailed data on life span (i.e. the age at the last observation of an individual in the study area), lifetime number of fledglings (i.e. the number of offspring fledglings over an individual's life), and lifetime number of recruits (i.e. an individual's lifetime number of offspring that had returned to the study area when ≥1 year old), of each breeding bird [37,40,41]. Furthermore, territory attractiveness rank was estimated annually for each breeding individual as the standardized mean occupation order of a territory in the two years flanking a given year. We also used data from 145 fledglings of the 1998 cohort. Molecular methods were used to verify the paternity and maternity of all chicks from 1987 and onwards [74,75]. For further details, see electronic supplementary material.

As an estimate of MHC-I diversity, we quantified the number of different MHC-I alleles per individual. We used tailor-made primers to amplify MHC-I exon 3, which encodes antigen-binding sites of MHC molecules [39], and sequenced the amplicons using 454-sequencing technology (for details, see electronic supplementary material). The sequencing data set included the samples from this study, plus 56 additional samples from a parallel study on the same species. In total, 351 samples were included, of which 42 were replicated, five were triplicated, and three were quadruplicated, resulting in a total of 412 sequenced amplicons. Data from 454-sequencing was demultiplexed using the software jMHC [76], filtered according to the method in Galan *et al.* [77], and inspected manually to remove low-quality and artificial sequences and non-functional alleles (see electronic supplementary material). We achieved a repeatability of the sequencing experiment of 0.94, estimated from the replicated samples. Finally, all sequences were identified by blasting against the NCBI database (http://blast.ncbi.nlm.nih.gov/Blast.cgi), and novel sequences given new names following the MHC standardized nomenclature [78].

All statistics were carried out in R version 3.1.2 [79]. Linear regression models were used to analyse sex-dependent effects of MHC-I diversity on three independent components of fitness for individual great reed warbler adults; *life span*, *offspring fledging success* (i.e. lifetime number of fledglings in models including life span as covariate), and *offspring recruitment success* (i.e. lifetime number of recruiting offspring in models including lifetime number of fledglings as covariate) (see electronic supplementary material for further details). These models followed the model design suggested by Gilks *et al.* [52]. All *p*-values are two-tailed. In figures, *offspring fledging success* was illustrated using the residual lifetime number of fledglings from a regression with life span, and similarly, *offspring recruitment success* was illustrated using the residual lifetime number of recruiting offspring from a regression with lifetime number of fledglings. To visualize a potentially nonlinear relationship between the MHC-I diversity and *offspring recruitment success*, we ran a cubic spline model of the effect of the MHC-I diversity on the residual lifetime number of recruiting offspring from a regression with lifetime number of fledglings. This model was run for each sex using the bs() function in the package ‘splines' in R [79].

The effect of the MHC-I diversity on the territory attractiveness rank was tested with linear mixed effects models that were run independently for each sex (see electronic supplementary material for details). The MHC-I diversity of males and females of 111 known social pairs were correlated using a Pearson's product–moment correlation test (in which each data point relates the values within a social pair). In cases of disassortative mating (i.e. if females with low MHC-I diversity preferentially mated with males with high MHC-I diversity and vice versa), the MHC-I diversities of pair mates should show a negative correlation. We used a GLMM model to test whether MHC-I diversity is a heritable trait by correlating the MHC-I diversity in offspring with the combined MHC-I diversity in parent pairs (the total number of different MHC-I alleles when combining the genotypes of the male and the female in a social pair). The combined MHC-I diversity in parent pairs was estimated using the package ‘MHCtools' in R [80]. This model included data from the fledglings of the 1998 cohort, their parents, and data from other related individuals in our data set (i.e. adult individuals that were born in previous years, and whose parents are both present in our data set); in total 176 offspring and 57 parent pairs. We tested whether MHC-I diversity in the offspring affected their recruitment success (i.e. becoming a recruit or not), using the data from the 145 fledglings of the 1998 cohort in a GLMM model. Details of these models are presented in the electronic supplementary material.

## Supplementary Material

Supplementary Information

## Data accessibility

Further details on the field methods are found in the electronic supplementary material. The dataset is available from the Dryad Digital Repository: http://dx.doi.org/10.5061/dryad.b321hf1 [81]. DNA sequences are available at GenBank (accession numbers: MH468831–MH469159).
