# Supplementary material for: Evidence for sexual conflict over major histocompatibility complex diversity in a wild songbird
Source: Proc Biol Sci. 2018 Aug 1;285(1884):20180841. doi: 10.1098/rspb.2018.0841 (PMC6111173; doi:10.1098/rspb.2018.0841)

Supplementary Information

Roved J., Hansson B., Tarka M., Hasselquist D., Westerdahl H. 2018 Evidence for sexual conflict over MHC diversity in a wild songbird. *Proc. R. Soc. B-Biological Sci.* (doi: 10.1098/rspb.2018.0841)

The study population

In the Lake Kvismaren study area 1984-2004, almost all (> 95 %) of the territorial great reed warbler males and breeding females were mist-netted and banded with unique combinations of color rings and one aluminum ring [1]. Visits were made to all territories on a daily basis (or occasionally every 2-3 days) throughout the breeding season, and we recorded the identity of each individual, arrival date, singing and breeding behaviors [1,2]. More than 95% of all nests were located and visited every third day from the start of nest building until fledging of the young or nest failure, and precise records were kept of all breeding attempts and their success [1]. Nestlings were banded with an aluminum ring and a color ring when 8-10 days old. All banded individuals that returned to the study area when ≥ 1 year old were identified, providing good estimates of life span and offspring recruitment success. None of the adults included in this study were observed in the study area after 2004. Extra-pair mating accounts for about 3% of the total number of offspring [3,4], and extra-pair offspring were assigned to their genetic parents in all analyses.

The territory attractiveness rank (in $year_n$) was calculated by taking the mean value of the relative date by which a territory was established by males the preceding ($year_{n-1}$) and subsequent years ($year_{n+1}$) [5]. These relative establishment ranks were standardized to $\bar{x} = 0$ and SD = 1 for each marsh (*i.e.,* sub-area of the study lake) and year [5]. The territory attractiveness rank values were inverted such that more attractive territories had higher ranks. To visualize the relationship between an individual's territory attractiveness ranks and MHC-I diversity in a two-dimensional plot, we adjusted the territory attractiveness ranks for each individual to an age of two years and used the mean of these age-adjusted territory attractiveness rank values for all years the individual was observed in Lake Kvismaren (mean territory rank). Further details on the field methods are found in references [1,2,6–8].

In our data set of adult great reed warblers, there were 173 successful and 17 unsuccessful first-time breeders. The recapture rate (among succeeding years) was 0.78 for successful first-time breeders, but 0.12 for unsuccessful first-time breeders. Individuals that were not recaptured may have died or dispersed to other breeding sites. Previous studies from our study population have indicated that individuals that fail their first breeding year are more likely to disperse to other breeding sites in following years [8,9]. Therefore, our fitness estimates for unsuccessful breeders are unlikely to be complete, and for this reason individuals that did not succeed in rearing any offspring (n = 17) were excluded from the analyses.

For the chicks of the 1998 cohort (n = 145) the offspring recruitment status (*i.e.* recruited or not) was used in statistical analyzes. There were 84 male fledglings of which 22 became recruits in Kvismaren and (in a few cases) in nearby lakes (*e.g.* in lake Tåkern and lake Hornborgasjön), and 61 female fledglings of which 11 became recruits in Kvismaren and (in a few cases) in nearby lakes.

DNA extraction, primers, PCR, and preparation of 454-sequencing

From each individual, we collected blood samples of 20-80 µl from the brachial or tarsus veins. The samples were suspended in 500-600 µl of SET-buffer (0.15 M NaCl, 0.05 M TRIS, 0.001 M ethylenediaminetetraacetate) and frozen at –20°C. Later, genomic DNA was isolated using standard phenol/chloroform-isoamylalcohol extraction [10], and the purified DNA was stored in 1× TE buffer and frozen at -50 or -80°C.

We used already published primers, forward (HNalla) TCCCCACAGGTCTCCACAC and reverse (HN46) ATCCCAAATTCCCACCCACCTT [11], that amplify a 262 bp region of exon 3, and have been designed based on MHC-I cDNAs in combination with intron 2 and intron 3 sequences from great reed warblers [11]. Tag sequences of six nucleotides (*i.e.,* molecular identifiers, MIDs) were added to the 5' end of the primers in order to create unique combinations of tagged primer pairs for each sample, enabling us to reassign sequences to samples after the 454 sequencing [12]. We used eight forward tags and 14 reverse tags. The samples from this study, plus 56 additional samples, were divided into four pools to be distributed over a 454 fiber-optic slide with a four-region gasket, using the same combinations of tagged primers in each pool. The total number of individuals included was 351, of which 42 samples were replicated, five were triplicated, and three were quadruplicated, resulting in a total number of 412 samples. In the PCR step, we added 16 blank controls. The PCR reaction mix for each sample consisted of 1.0 µl DNA solution (20 ng/µl in ddH_2O), 1.2 µl tagged forward primer (2.5 µM), 1.2 µl tagged reverse primer (2.5 µM), 9.5 µl Multimix (Qiagen N.V., Venlo, Netherlands), and 2.1 µl ddH_2O. PCRs were initiated by a 15 minutes heating phase at 95°C, followed by 30 cycles of 30 sec denaturation at 95°C, 90 sec annealing at 65°C, 60 sec elongation at 72°C, and ended with 10 min at 72°C. The PCR results were confirmed by gel electrophoresis in 2% agarose gels, and samples were then pooled eight by eight and cleaned using the MinElute PCR Purification Kit (Qiagen N.V., Venlo, Netherlands). The DNA concentration of each pool was measured on a Nanodrop 2000C spectrophotometer, and finally the samples pooled again to equal concentrations and delivered for 454-sequencing. Sequencing was done in a Roche 454 GS FLX (F. Hoffmann-La Roche AG, Basel, Switzerland) following the manufacturer's instructions at the Department of Biology at Lund University.

Filtering and screening of 454-sequence data

The 454-sequencing data contained 97,900 unique sequence variants and the total coverage was 551,258 reads. The data was demultiplexed using the software jMHC [13]. Sequences with no reads or to which tags had not been successfully assigned were filtered out in this step. Identical sequences were detected and merged using the web-based freewares Seqeqseq (http://mbio-serv2.mbioekol.lu.se/apps/seqeqseq.html) and mergeMatrix (http://mbio-serv2.mbioekol.lu.se/apps/mergeMatrix.html). Sequences with < 3 reads across all samples were removed, reducing the data set to 16,587 variants with 455,718 reads among 412 samples. Further filtering was performed in the web-based freeware popMatrix (http://mbio-serv2.mbioekol.lu.se/apps/popMatrix.html), which is designed to filter data from high-throughput sequencing following the principles in Galan *et al.* (2010) [14]. In this process, samples with < 240 reads and sequences with a relative abundance of < 0.012 within each sample were removed. The threshold for the first filter was estimated from the distribution of the number of reads per sample. The threshold for the second filter was estimated from the distribution of within-sample frequencies of all sequences, following Galan *et al.* (2010) [14], and verified by matching the replicated samples in the data set. After this filtering step, 511

sequence variants remained with 311,108 reads. 3 samples with < 240 reads had been removed. The remaining sequences were aligned and inspected manually using BioEdit (version 7.1.11). Sequences that contained stop codons or indels obstructing the reading frame, other non-functional sequences and known pseudogene alleles were removed from the data set at this point. PCR chimeras and single nucleotide substitution errors were also identified and removed. After final filtering and screening, there were 329 sequence variants in the data set. The replicated samples in the data set were merged so that any sequence that remained in any one sample after filtering and screening (unique variants) got to stay in the data set. One genotyped sample was excluded from further analyses because it could not be assigned to an individual in the database. The proportions of unique variants (PUV) between replicated samples were calculated and the repeatability of the total sequencing reaction was calculated as [1 – mean PUV] = 0.94.

All DNA sequences were identified by blasting against the NCBI database (http://blast.ncbi.nlm.nih.gov/Blast.cgi) and novel sequences given new names following the MHC standardized nomenclature [12].

Statistical models

The effects of MHC-I diversity on life span

The linear regression model included *life span* as dependent variable, MHC-I diversity (*i.e.*, the number of different MHC-I alleles per individual) and MHC-I diversity squared as explanatory variables, sex as a fixed factor, and the interactions MHC-I diversity × sex, and MHC-I diversity squared × sex. The model was simplified step by step by removing the least significant terms. Analyses of variance and changes in the Akaike information criterion (AIC) were used to confirm that each simplification step improved the fit of the model. Histograms of the residuals from the *life span* models showed that they did not follow a normal distribution (Fig. S5 a, b), and we therefore ran the models also as log-linked generalized linear models with negative binomial error distribution using the package 'MASS' in R [15]. The results from these confirmed the results obtained with the Gaussian linear regression models (Table S7 a-c).

The effects of MHC-I diversity on offspring fledging success

We tested the effect of MHC-I diversity on the lifetime number of fledglings with a linear regression model that included life span as covariate (*i.e.*, an analysis of *offspring fledging success*). The other terms in the model were specified as in the model on *life span* (above) and the model was simplified step by step by removing the least significant terms. Analyses of variance and changes in AIC were used to confirm that each simplification step improved the fit of the model. The residuals from these models were normally distributed (Fig. S6 a, b).

For illustrative purposes, residuals from a linear model between lifetime number of fledglings and life span for each sex (Table S8) were saved as a new variable, that explains *offspring fledging success*, and this was correlated with MHC-I diversity using a linear regression model.

The effects of MHC-I diversity on offspring recruitment success

We tested the effect of MHC-I diversity on the lifetime number of recruiting offspring with a linear regression model including lifetime number of fledglings as covariate (*i.e.*, an analysis of *offspring recruitment success*). The other terms in the model were specified as in the model on *life span* (above) and and the model was simplified step by step by removing the least significant terms. Analyses of variance and changes in AIC were used to confirm that each simplification

step improved the fit of the model. The residuals from this model are normally distributed (Fig. S7). The models were also run independently for each sex, testing the effect of MHC-I diversity on the lifetime number of recruiting offspring with lifetime number of fledglings as a covariate. The residuals from these models are normally distributed (Fig. S8 a, b).

In order to obtain selection gradients [16], we calculated the relative lifetime number of recruiting offspring of each individual by dividing the values with the mean of all individuals in our data set. We then repeated the final models with relative lifetime number of recruiting offspring as response variable (with lifetime number of fledglings as covariate).

For illustrative purposes, residuals from a linear model between lifetime number of recruiting offspring and lifetime number of fledglings for each sex (Table S9) were saved as a new variable, that explains *offspring recruitment success*, and this was correlated with the number of different MHC-I alleles using a linear regression model.

The effects of MHC-I diversity on territory attractiveness rank
The effect of the MHC-I diversity on the territory attractiveness rank was tested with linear mixed effects models using the package 'lme4' in R [17]. The models were conducted independently for each sex and had yearly recorded values of the territory attractiveness rank as response variable, the MHC-I diversity as explanatory variable, and age given individual as random factor. Two-tailed p-values were approximated from a t-distribution given the degrees of freedom of the parameter estimates.

Parent-offspring regression of MHC-I diversity
The model included MHC-I diversity in the offspring as response variable, the combined MHC-I diversity in the parent pairs (*i.e.*, the sum of the number of alleles in each parent subtracting the number of shared alleles) as explanatory variable, and the pair ID of the parents as random factor. The combined MHC-I diversity in the parent pairs takes into account that the MHC-I diversity in a parent pair may be smaller than the sum of the MHC-I diversity from each parent because certain alleles may be shared between the parents. The combined MHC diversity in parent pairs was estimated using the package 'MHCtools' in R [18]. The model was run with the package 'lme4' in R [17]. A two-tailed p-value was approximated from a t-distribution given a number of degrees of freedom estimated with Kenward-Roger approximation using the package 'pbkrtest' in R [19].

The effects of MHC-I diversity in offspring on their recruitment success
We tested whether MHC-I diversity in the offspring affected their recruitment success using a generalized linear mixed model with logit link function. The model had offspring recruitment status (*i.e.*, recruited or not) as response variable, MHC-I diversity as explanatory variable, and included nest ID as a random factor. We also tested if offspring sex affected the relationship between their MHC-I diversity and recruitment success, by repeating the model including sex as a fixed factor and the interaction MHC-I diversity × sex. These models were run with the package 'lme4' in R [17] using the data from the 145 fledglings of the 1998 cohort.

Supplementary Results
First, we analyzed whether MHC-I diversity affected *life span*, when including sex as a fixed factor. The full quadratic model showed no significance for the interactions MHC-I diversity × sex and MHC-I diversity squared × sex (Table S1 a), and AIC decreased from 716.78 to 713.01

when they were removed from the model. This was confirmed by an anova comparing the models with or without the interaction terms (p = 0.9). In the model without the interaction terms, sex was least significant (Table S1 b), and removing that term from the model was confirmed by confirmed by a drop from 713.01 to 711.10 in AIC and by comparing the two models with an anova (p = 0.77). In the model without sex, MHC-I diversity was the least significant term (Table S1 c) and removing that term from the model reduced the AIC from 711.10 to 709.44 and was confirmed by an anova (p = 0.56). The final model included only MHC-I diversity squared and showed no significant effect (quadratic regression, b = 0.00037, SE = 0.0017, t = 0.22, d.f. = 169, p = 0.83; Table S1 d).

Second, we analyzed the effect of MHC-I diversity on *offspring fledging success*. This model had the lifetime number of fledglings as dependent variable, including life span as covariate, and sex as a fixed factor. The full quadratic model showed no significance for the interactions MHC-I diversity × sex and MHC-I diversity squared × sex (Table S2 a), and AIC was reduced from 1152.56 to 1149.36 when they were removed from the model. This was confirmed by an anova comparing the models with or without the interaction terms (p = 0.68). In the model without the interaction terms, MHC-I diversity was the least significant term (Table S2 b), and an anova confirmed removing that term from the model (p = 0.86), and this was also confirmed by a drop in AIC from 1149.36 to 1147.40. The final model included life span, MHC-I diversity squared, sex, and the interaction life span × sex. It showed no significant effect of MHC-I diversity squared (quadratic regression, b = -0.0016, SE = 0.006, t = -0.26, d.f. = 166, p = 0.80; Table S2 c).

Third, we tested the effect of MHC-I diversity on *offspring recruitment success*. This model had the lifetime number of recruiting offspring as dependent variable and lifetime number of fledglings as covariate, including sex as a fixed factor. The full quadratic model showed no significance for the interactions MHC-I diversity × sex and MHC-I diversity squared × sex (Table S3 a), but AIC increased from 588.24 to 595.03 when they were removed, so we retained them in the model. The linear term MHC-I diversity was less significant than MHC-I diversity squared and removing MHC-I diversity and the interaction MHC-I diversity × sex from the model reduced the AIC from 588.24 to 586.35. An anova model confirmed removing those terms (p = 0.37). The final model included lifetime number of fledglings, MHC-I diversity squared, sex, and the interactions lifetime number of fledglings × sex and MHC-I diversity squared × sex. It showed a highly significant for the interaction between MHC-I diversity squared and sex (quadratic regression, b = 0.0071, SE = 0.0023, t = 3.04, d.f. = 165, p = 0.0028; Fig. 1; Table 1). The model was repeated for males and females separately. In males, MHC-I diversity squared was the least significant term (Table S3 b), and an anova confirmed removing that from the model (p = 0.68). This was also confirmed by a reduction in AIC from 240.09 to 238.27. The final model included lifetime number of fledglings and MHC-I diversity and showed a significant effect of MHC-I diversity (linear regression, b = 0.084, SE = 0.041, t = 2.04, d.f. = 73, p = 0.045; Fig. 1; Table 1). In females, MHC-I diversity was the least significant term (Table S3 c), and an anova confirmed removing that from the model (p = 0.26). This was also confirmed by a reduction in AIC-values from 345.04 to 344.39. The final model included lifetime number of fledglings and MHC-I diversity squared and showed a significant effect of MHC-I diversity squared (quadratic regression, b = -0.0044, SE = 0.0018, t = -2.41, d.f. = 92, p = 0.018; Fig. 1; Table 1).

References

1. Bensch S, Hasselquist D, Nielsen B, Hansson B. 1998 Higher fitness for philopatric than for immigrant males in a semi-isolated population of great reed warblers. *Evolution (N. Y).* **52**, 877–883. (doi:10.2307/2411282)
2. Hasselquist D, Bensch S. 1991 Trade-off between mate guarding and mate attraction in the polygynous great reed warbler. *Behav. Ecol. Sociobiol.* **28**, 187–193.
3. Hasselquist D, Bensch S, Von Schantz T. 1995 Low frequency of extrapair paternity in the polygynous great reed warbler, Acrocephalus arundinaceus. *Behav. Ecol.* **6**, 27–38. (doi:10.1093/beheco/6.1.27)
4. Hasselquist D, Bensch S, Von Schantz T. 1996 Correlation between male song repertoire, extra-pair paternity and offspring survival in the great reed warbler. *Nature* **381**, 229–232. (doi:10.1038/381229a0)
5. Bensch S. 1996 Female mating status and reproductive success in the great reed warbler: Is there a potential cost of polygyny that requires compensation? *J. Anim. Ecol.* **65**, 283–296. (doi:10.2307/5875)
6. Tarka M, Akesson M, Hasselquist D, Hansson B. 2014 Intralocus sexual conflict over wing length in a wild migratory bird. *Am. Nat.* **183**, 62–73. (doi:10.1086/674072)
7. Hasselquist D. 1998 Polygyny in great reed warblers: A long-term study of factors contributing to male fitness. *Ecology* **79**, 2376–2390. (doi:10.1890/0012-9658(1998)079[2376:pigrwa]2.0.co;2)
8. Bensch S, Hasselquist D. 1991 Territory infidelity in the polygynous great reed warbler Acrocephalus arundinaceus: The effect of variation in territory attractiveness. *J. Anim. Ecol.* **60**, 857–871. (doi:10.2307/5418)
9. Hansson B, Bensch S, Hasselquist D, Nielsen B. 2002 Restricted dispersal in a long-distance migrant bird with patchy distribution, the great reed warbler. *Oecologia* **130**, 536–542. (doi:10.1007/s00442-001-0831-2)
10. Sambrook J, Fritsch EF, Maniatis T. 1989 *Molecular cloning: a laboratory manual*. 2nd edn. Cold Spring Harbor: Cold Spring Harbor Laboratory Press.
11. Westerdahl H, Wittzell H, von Schantz T, Bensch S. 2004 MHC class I typing in a songbird with numerous loci and high polymorphism using motif-specific PCR and DGGE. *Heredity (Edinb).* **92**, 534–542. (doi:10.1038/sj.hdy.6800450)
12. Klein J *et al.* 1990 Nomenclature for the major histocompatibility complexes of different species - a proposal. *Immunogenetics* **31**, 217–219.
13. Stuglik MT, Radwan J, Babik W. 2011 jMHC: Software assistant for multilocus genotyping of gene families using next-generation amplicon sequencing. *Mol. Ecol. Resour.* **11**, 739–742. (doi:10.1111/j.1755-0998.2011.02997.x)
14. Galan M, Guivier E, Caraux G, Charbonnel N, Cosson J-F. 2010 A 454 multiplex sequencing method for rapid and reliable genotyping of highly polymorphic genes in large-scale studies. *BMC Genomics* **11**. (doi:10.1186/1471-2164-11-296)
15. Venables WN, Ripley BD. 2002 *Modern applied statistics with S.* Fourth. New York: Springer. See http://www.stats.ox.ac.uk/pub/MASS4.
16. Lande R, Arnold SJ. 1983 The measurement of selection on correlated characters. *Evolution (N. Y).* **37**, 1210–1226. (doi:10.2307/2408842)
17. Bates D, Mächler M, Bolker B, Walker S. 2014 lme4: Linear Mixed-Effects Models using Eigen and S4.
18. Roved J. 2017 MHCtools: Analysis of MHC Data in Non-Model Species. *R Packag.* (doi:10.13140/RG.2.2.19711.71847)
19. Halekoh U, Højsgaard S. 2014 A Kenward-Roger approximation and parametric bootstrap methods for tests in linear mixed models - The R package pbkrtest. *J. Stat. Softw.* **59**, 1–32. (doi:10.18637/jss.v059.i09)

Table S1 Linear models of the effect of MHC-I diversity (*i.e.*, the number of different MHC-I alleles per individual) and sex on *life span*. (a) The full model including MHC-I diversity squared, MHC-I diversity, sex, and the interactions MHC-I diversity squared × sex and MHC-I diversity × sex, (b) without the interactions, (c) without sex, and (d) without the linear term MHC-I diversity. See Supplementary Results for details on the model selection.

a

	Estimate	Std. error	t-value	P-value
**(Intercept)**	3.84	3.11	1.24	0.22
**(MHC-I diversity)^2**	0.0039	0.017	0.23	0.81
**MHC-I diversity**	-0.086	0.46	-0.19	0.85
**Sex (male)**	1.43	4.11	0.35	0.73
**(MHC-I diversity)^2 × Sex (male)**	0.0043	0.021	0.20	0.84
**MHC-I diversity × Sex (male)**	-0.16	0.60	-0.27	0.79

b

	Estimate	Std. error	t-value	P-value
**(Intercept)**	4.48	2.00	2.25	0.026
**(MHC-I diversity)^2**	0.0061	0.010	0.60	0.55
**MHC-I diversity**	-0.16	0.29	-0.57	0.57
**Sex (male)**	0.088	0.29	0.30	0.77

c

	Estimate	Std. error	t-value	P-value
**(Intercept)**	4.54	1.98	2.29	0.023
**(MHC-I diversity)^2**	0.0062	0.010	0.61	0.54
**MHC-I diversity**	-0.17	0.29	-0.58	0.56

d

	Estimate	Std. error	t-value	P-value
**(Intercept)**	3.40	0.35	9.64	<0.0001
**(MHC-I diversity)^2**	0.00037	0.0017	0.22	0.83

Table S2 Linear models of the effect of MHC-I diversity (*i.e.*, the number of different MHC-I alleles per individual) and sex on *offspring fledgling success*. (a) The full model including life span, MHC-I diversity squared, MHC-I diversity, sex, and the interactions life span × sex, MHC-I diversity squared × sex, and MHC-I diversity × sex, (b) without the interactions MHC-I diversity squared × sex and MHC-I diversity × sex, and (c) without the linear term MHC-I diversity. See Supplementary Results for details on the model selection.

a

	Estimate	Std. error	t-value	P-value
**(Intercept)**	-2.49	11.13	-0.22	0.82
**Life span**	2.84	0.33	8.65	<0.0001
**(MHC-I diversity)^2**	-0.0073	0.060	-0.12	0.90
**MHC-I diversity**	0.31	1.65	0.19	0.85
**Sex (male)**	1.05	14.92	0.070	0.94
**Life span × Sex (male)**	2.35	0.61	3.85	0.00017
**(MHC-I diversity)^2 × Sex (male)**	0.0050	0.076	0.065	0.95
**MHC-I diversity × Sex (male)**	-0.44	2.13	-0.21	0.84

b

	Estimate	Std. error	t-value	P-value
**(Intercept)**	-0.76	7.22	-0.11	0.92
**Life span**	2.85	0.33	8.70	<0.0001
**(MHC-I diversity)^2**	-0.0081	0.036	-0.22	0.82
**MHC-I diversity**	0.19	1.03	0.18	0.86
**Sex (male)**	-3.95	2.37	-1.67	0.10
**Life span × Sex (male)**	2.36	0.61	3.89	0.00015

c

	Estimate	Std. error	t-value	P-value
**(Intercept)**	0.52	1.72	0.30	0.76
**Life span**	2.85	0.33	8.72	<0.0001
**(MHC-I diversity)^2**	-0.0016	0.0061	-0.26	0.80
**Sex (male)**	-3.93	2.36	-1.67	0.10
**Life span × Sex (male)**	2.35	0.60	3.89	0.00014

Table S3 Linear models of the effect of MHC-I diversity (*i.e.*, the number of different MHC-I alleles per individual) and sex on *offspring recruitment success*. (a) The full model including lifetime number of fledglings, MHC-I diversity squared, MHC-I diversity, sex, and the interactions lifetime number of fledglings × sex, MHC-I diversity squared × sex, and MHC-I diversity × sex. (b) The same model repeated for males only, and (c) for females only.

a

	Estimate	Std. error	t-value	P-value
**(Intercept)**	-2.21	2.13	-1.04	0.30
**Fledglings**	0.21	0.019	11.37	<0.0001
**(MHC-I diversity)^2**	-0.019	0.011	-1.62	0.11
**MHC-I diversity**	0.40	0.32	1.25	0.21
**Sex (male)**	0.48	2.83	0.17	0.87
**Fledglings × Sex (male)**	-0.068	0.022	-3.04	0.0027
**(MHC-I diversity)^2 × Sex (male)**	0.015	0.015	1.06	0.29
**MHC-I diversity × Sex (male)**	-0.22	0.41	-0.54	0.59

b

Males	Estimate	Std. error	t-value	P-value
**(Intercept)**	-1.73	1.60	-1.08	0.28
**Fledglings**	0.14	0.011	13.32	<0.0001
**(MHC-I diversity)^2**	-0.0032	0.0077	-0.41	0.68
**MHC-I diversity**	0.17	0.22	0.78	0.44

c

Females	**Estimate**	**Std. error**	**t-value**	**P-value**
**(Intercept)**	-2.21	2.34	-0.94	0.35
**Fledglings**	0.21	0.021	10.35	<0.0001
**(MHC-I diversity)^2**	-0.019	0.013	-1.48	0.14
**MHC-I diversity**	0.40	0.35	1.14	0.26

Table S4 Selection gradient estimates. Regression models of the effect of MHC-I diversity (*i.e.*, the number of different MHC-I alleles per individual) and sex on the relative lifetime number of recruiting offspring (*i.e.*, lifetime number of recruiting offspring divided by the population mean) accounting for lifetime number of fledglings.

	**Estimate**	**Std. error**	**t-value**	**P-value**
Both sexes				
**(Intercept)**	0.22	0.20	1.13	0.26
**Fledglings**	0.11	0.0098	11.37	<0.0001
**(MHC-I diversity)^2**	-0.0023	0.00087	-2.65	0.0088
**Sex (male)**	-0.49	0.29	-1.67	0.10
**Fledglings × Sex (male)**	-0.036	0.012	-3.08	0.0025
**(MHC-I diversity)^2 × Sex (male)**	0.0038	0.0012	3.04	0.0028
Females				
**(Intercept)**	0.22	0.22	1.03	0.31
**Fledglings**	0.11	0.011	10.34	<0.0001
**(MHC-I diversity)^2**	-0.0023	0.00096	-2.41	0.018
Males				
**(Intercept)**	-0.59	0.32	-1.85	0.068
**Fledglings**	0.076	0.0056	13.39	<0.0001
**MHC-I diversity**	0.044	0.022	2.04	0.045

Table S5 Correlation between the MHC-I diversity of offspring (n = 176) and the combined MHC-I diversity of their parents (n = 57 parent pairs).

	Estimate	Std. Error	t-value	P-value
**(Intercept)**	4.110	0.994	4.14	<0.001
**Combined MHC-I diversity in parents**	0.393	0.042	9.47	<0.0001

Table S6 (a) Test of the effect of MHC-I diversity in offspring on their recruitment success (glmm; n = 145; deviance = 153.8; res. d.f. = 142). (b) Test of the effect of sex and MHC-I diversity in offspring on their recruitment success (glmm; n = 145; deviance = 152.1; res. d.f. = 140).

a

	Estimate	Std. Error	z-value	P-value
**(Intercept)**	-0.075	0.885	-0.09	0.93
**MHC-I diversity**	-0.087	0.067	-1.31	0.19

b

	Estimate	Std. Error	z-value	P-value
**(Intercept)**	2.104	2.677	0.79	0.43
**MHC-I diversity**	-0.206	0.202	-1.02	0.31
**Sex (male)**	-1.643	1.893	-0.87	0.39
**MHC-I diversity × Sex (male)**	0.091	0.140	0.65	0.52

Table S7 Generalized linear models (with neg. binomial error distribution) of the effect of MHC-I diversity (*i.e.*, the number of different MHC-I alleles per individual) and sex on *life span*. (a) The full model including MHC-I diversity, sex, and the interaction MHC-I diversity × sex, (b) without the interaction, and (c) without sex.

a

	Estimate	Std. error	t-value	P-value
**(Intercept)**	1.15	0.26	4.37	<0.0001
**MHC-I diversity**	0.0062	0.019	0.32	0.75
**Sex (male)**	0.16	0.38	0.41	0.68
**MHC-I diversity × Sex (male)**	-0.0097	0.028	-0.35	0.73

b

	Estimate	Std. error	t-value	P-value
**(Intercept)**	1.21	0.19	6.27	<0.0001
**MHC-I diversity**	0.0015	0.014	0.11	0.91
**Sex (male)**	0.027	0.085	0.32	0.75

c

	Estimate	Std. error	t-value	P-value
**(Intercept)**	1.22	0.19	6.45	<0.0001
**MHC-I diversity**	0.0016	0.014	0.12	0.91

Table S8 Linear regression model of the lifetime number of fledglings on life span with sex as a fixed factor and including the interaction life span × sex.

	Estimate	Std. Error	t-value	P-value
**(Intercept)**	0.235	1.315	0.18	0.86
**Life span**	2.846	0.325	8.75	<0.0001
**Sex (male)**	-3.948	2.350	-1.68	0.095
**Life span × Sex (male)**	2.353	0.602	3.91	<0.001

Table S9 Linear regression model of the lifetime number of recruiting offspring on the lifetime number of fledglings with sex as a fixed factor and including the interaction lifetime number of fledglings × sex.

	Estimate	Std. Error	t-value	P-value
**(Intercept)**	-0.363	0.235	-1.54	0.12
**Fledglings**	0.209	0.019	10.96	<0.0001
**Sex (male)**	0.406	0.338	1.20	0.23
**Fledglings × Sex (male)**	-0.067	0.023	-2.92	0.0040

Fig. S1 Distribution of the number of different MHC-I alleles (a) in adult male and female and (b) in nestling male and female great reed warblers.

a

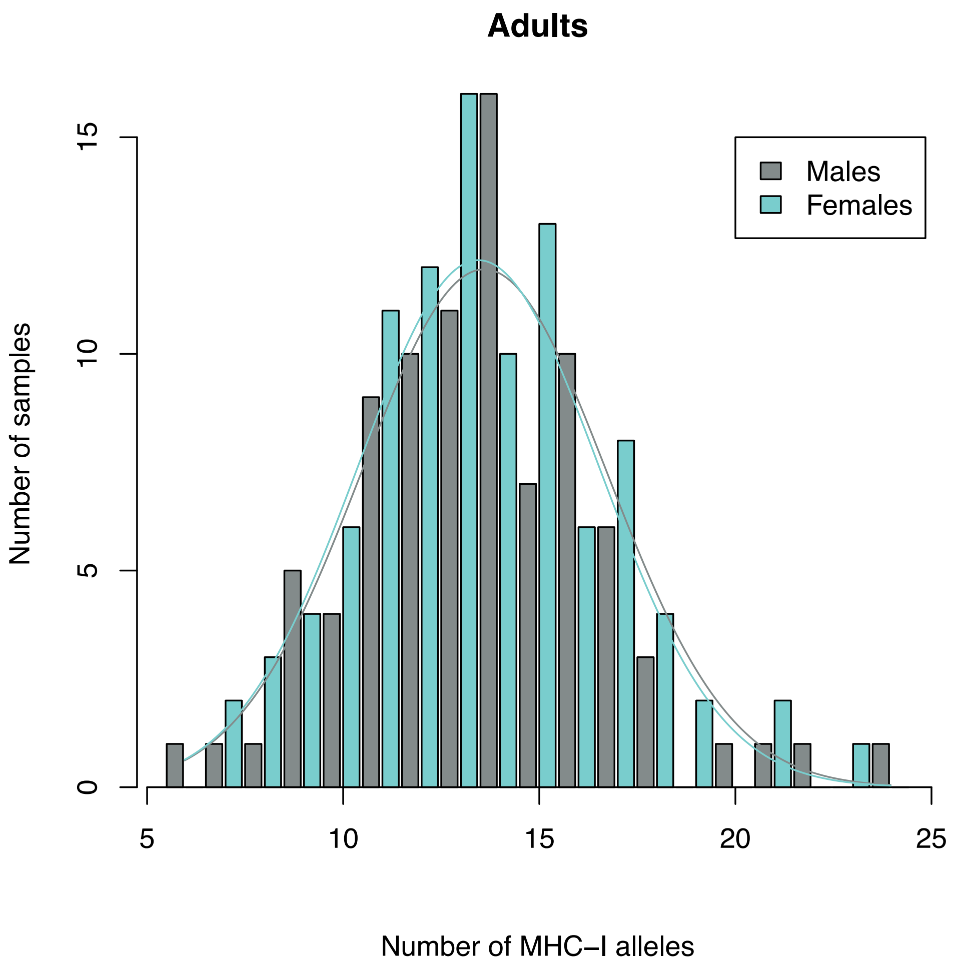

b

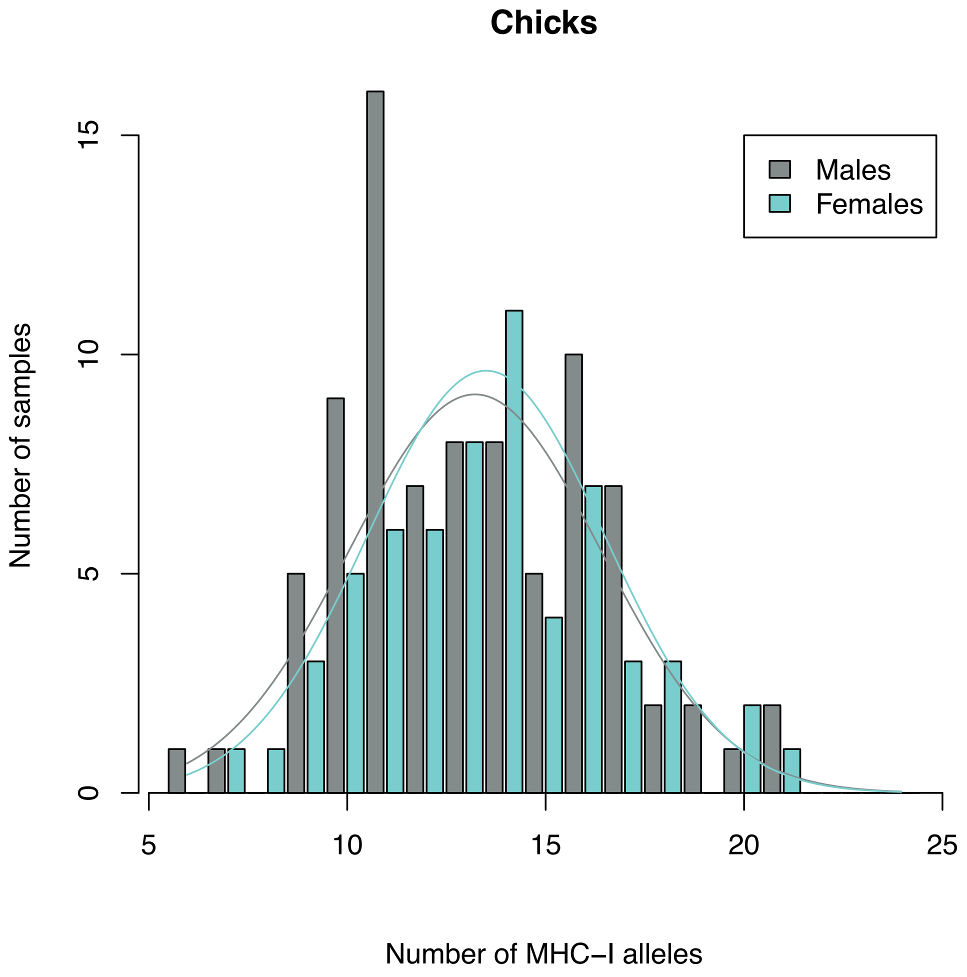
Chicks
Males
Females
Number of samples
0
5
10
15
5
10
15
20
25
Number of MHC−I alleles

Fig. S2 The relationship between *life span* and MHC-I diversity (*i.e.*, the number of different MHC-I alleles per individual). Females are shown with open circles and males are shown with black triangles. The line shows the prediction from a regression of life span on MHC-I diversity squared. Note: Jitter was added to life span and number of different MHC-I alleles to distinguish individual data points.

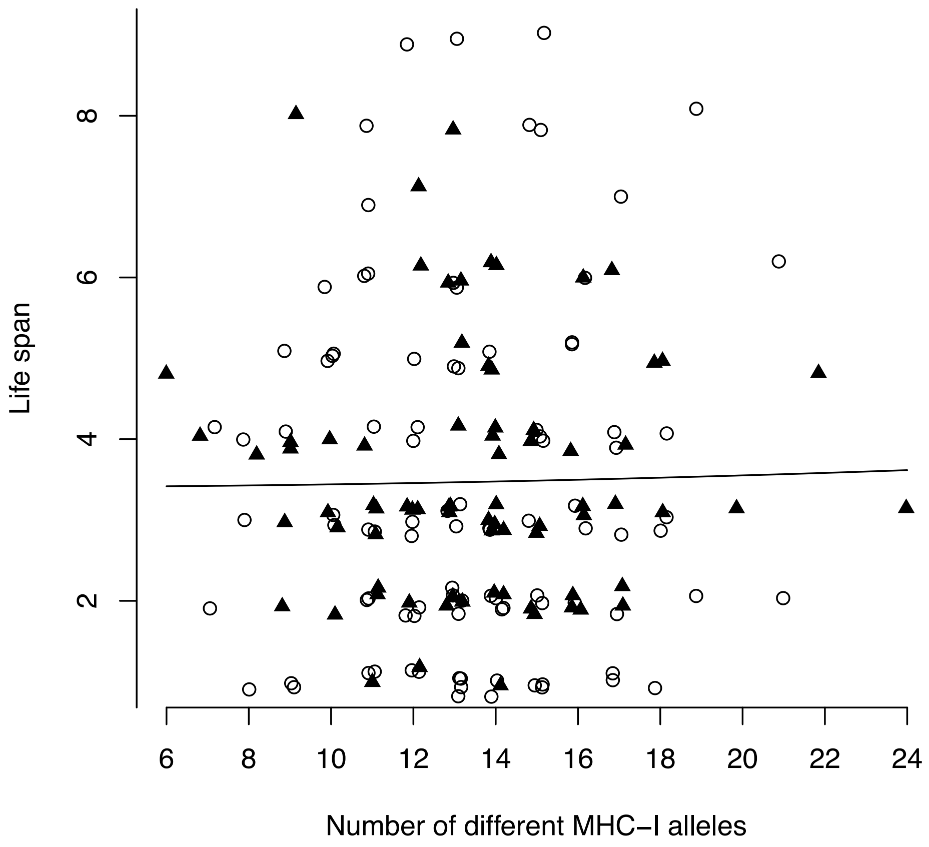

Fig. S3 The relationship between MHC-I diversity (*i.e.*, the number of different MHC-I alleles per individual) and the *offspring fledging success* (*i.e.*, the lifetime number of fledglings accounting for life span). Females are shown with open circles and males are shown with black triangles. The line shows the prediction from a regression of the offspring fledging success on the MHC-I diversity squared with sex as a fixed factor. *Offspring fledging success* is illustrated using the residual lifetime number of fledglings from a regression with life span. Note: Jitter was added to the number of different MHC-I alleles to distinguish individual data points.

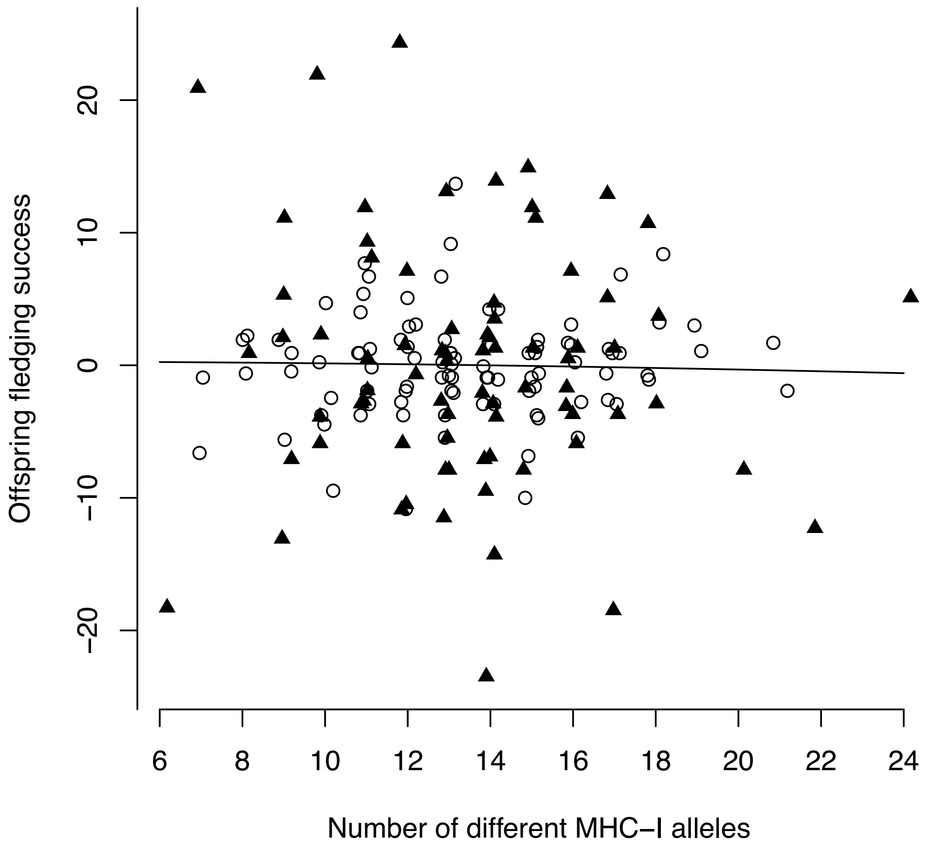

Fig. S4 The relationship between the mean territory rank and MHC-I diversity (*i.e.*, the number of different MHC-I alleles per individual) in adult female great reed warblers with predicted values from a linear regression model. The relationship is illustrated using the lifetime mean of age-standardized territory attractiveness rank values. Note: Jitter was added to the number of different MHC-I alleles to distinguish individual data points.

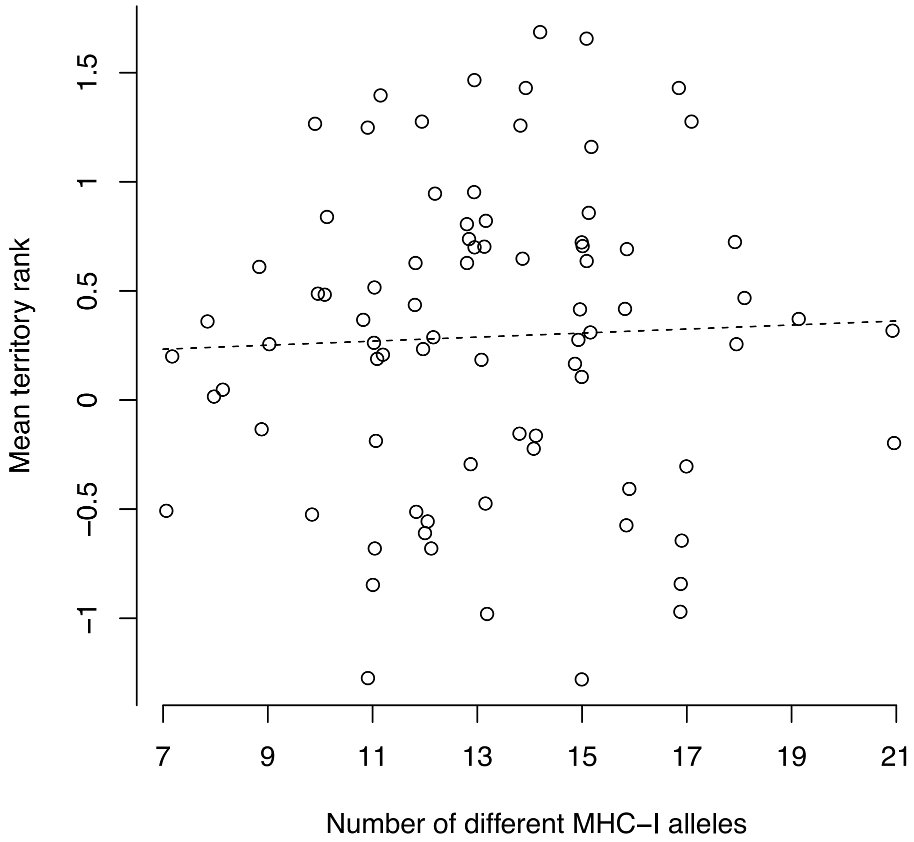

Fig. S5 Histograms of the residuals from linear regression models of lifespan on MHC-I diversity (see text for model details).

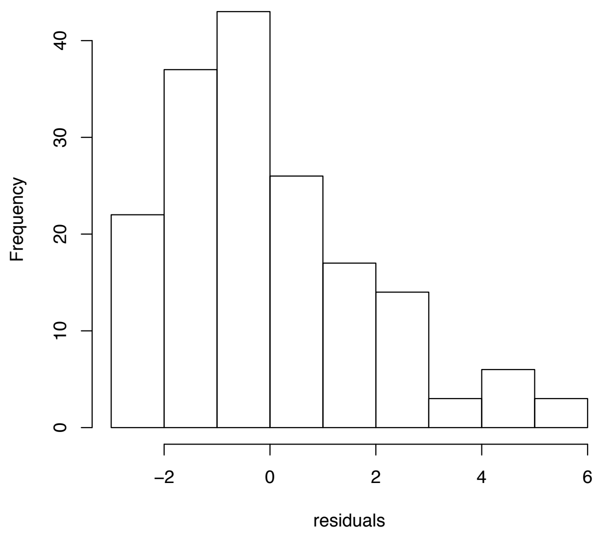

Fig. S6 Histograms of the residuals from linear regression models of the lifetime number of fledglings on MHC-I diversity with lifespan as covariate (see text for model details).

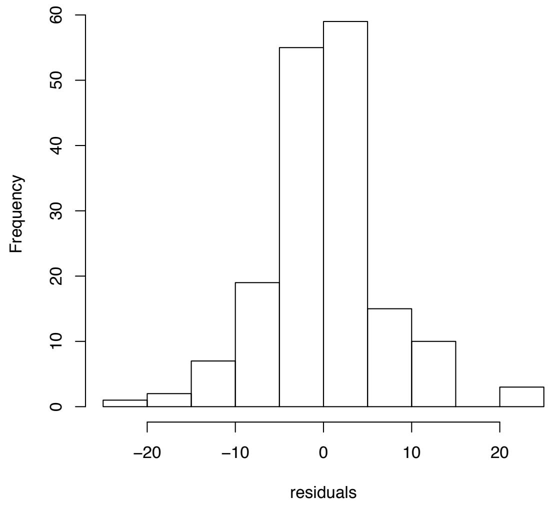

Fig. S7 Histogram of the residuals from the regression model of the lifetime number of recruiting offspring on MHC-I diversity with the lifetime number of fledglings as covariate (see text for model details).

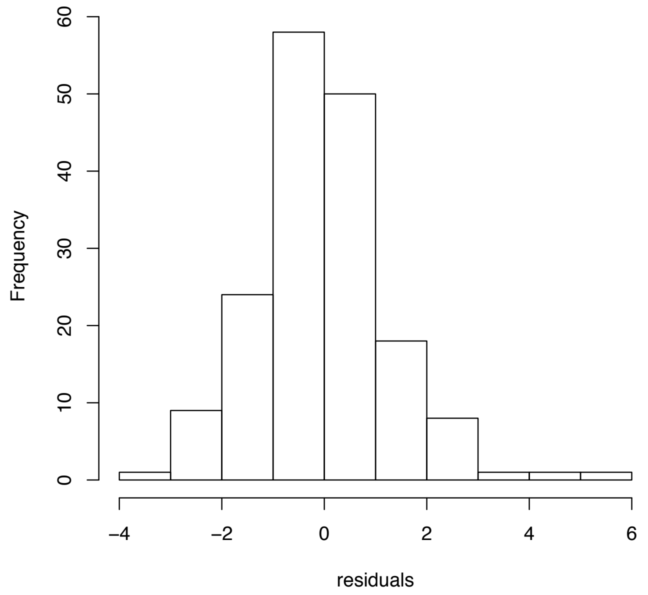

Fig. S8 Histograms of the residuals from the linear regression models of the lifetime number of recruiting offspring on MHC-I diversity with lifetime number of fledglings as covariate in males (a) and females (b) (see text for model details).

a

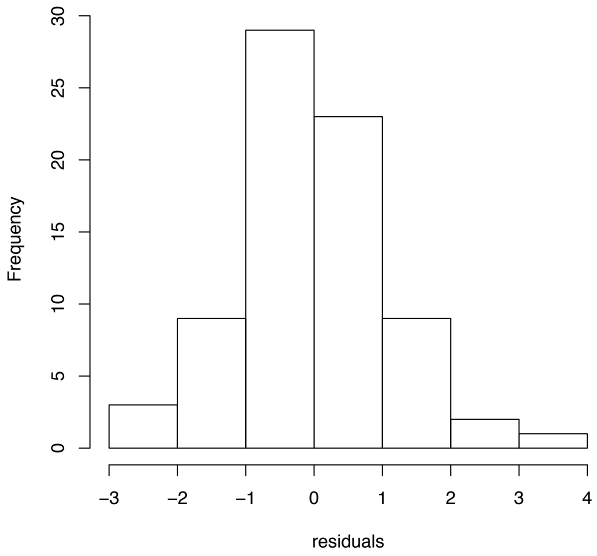

b

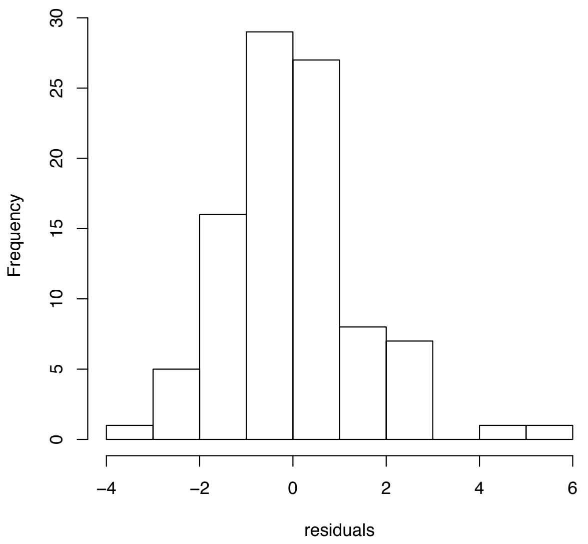

Supplement: Supplementary Information [file rspb20180841supp1.pdf]
